# Hematological diseases and the heart

**DOI:** 10.55730/1300-0144.5929

**Published:** 2024-12-02

**Authors:** Ferda CAN

**Affiliations:** Division of Hematology, Department of Internal Medicine, Ankara Bilkent City Hospital, Ankara, Turkiye

**Keywords:** Hematological disease, heart, cardiac involvement

## Abstract

The heart, as the main part of the circulatory system, is one of the organs frequently affected by hematological diseases. Cardiac problems may develop during the course and treatment of benign and malignant hematological diseases. This review study was conducted based on recent information from the literature and manifestations of cardiac involvement in hematology were summarized. Determining appropriate cardiac approaches in cases of hematological diseases with relevant knowledge and experience regarding the development of cardiac involvement in pathophysiological conditions has an important role in the diagnosis and treatment processes of patients. This review provides relevant information about hematological diseases and the heart, and key points that will help in the diagnosis and treatment process are presented.

## 1. Introduction

As the heart is a main part of both the circulatory system and the vascular system, cardiac involvement is frequently seen in hematological conditions. In addition to hematological diseases with direct cardiac involvement, such as amylolysis, lymphoma in the form of mass lesions, leukemias, or hypereosinophilic syndrome, there are types of cardiac involvement due to conditions such as endothelial damage, intravascular events, and interference with the coagulation cascade. Variables such as the frequency of cardiac involvement and the sex distribution of patients are associated with the underlying hematological disease. In addition to classical chemotherapy drugs, treatment-related cardiotoxicity has been seen with new treatment modalities such as immunotherapy and cellular therapies, which are increasingly used in recent years. Cardiotoxicity can occur in many forms, ranging from asymptomatic to fatal conditions. In cases of some conditions, treatment discontinuation may be required due to these side effects; thus, the patient’s hematological disease may be severely affected. Cardiological conditions observed together with hematological conditions are examined in the following sections of this review.

## 2. Hemoglobinopathies and the heart

Heart involvement is the leading cause of death in patients with beta thalassemia and sickle cell disease. Heart involvement is also the main prognostic organ involvement directly affecting mortality.

Beta thalassemia, a genetic disorder caused by the coding of the beta-globin gene of hemoglobin (*HBB*), is characterized by hemolytic anemia and ineffective erythropoiesis. Beta thalassemia major, which is the most clinically important subtype, is diagnosed in early infancy, often within the first 2 years of life, with severe anemia requiring transfusion [1]. Although mortality rates and iron overload-related complications have decreased in this patient group in recent years with the development of transfusion medicine, widespread use of transfusions, allogeneic stem cell transplantation, developments in gene therapy, and new medical treatments including iron chelators, cardiac involvement still exerts significant effects in terms of morbidity and remains one of the main causes of mortality in beta thalassemia patients [2–4].

Sickle cell disease is an inherited disease characterized by the presence of sickle-shaped erythrocytes in circulation with an increase in hemoglobin S (HbS) as a result of a nucleotide change in the beta-globin gene. HbS has a soluble structure and can be dissolved under appropriate oxygen conditions, but when oxygen levels decrease, HbS polymerizes and these polymers cause the sickling of erythrocytes. In turn, the sickle cells cause vascular occlusion and tissue hypoxia leading to painful crises and organ infarctions. These erythrocytes also cause a chronic hemolytic process, as in beta thalassemia. The leading cause of death in this patient group is cardiac disease [5].

The pathological mechanisms causing cardiac disease in hemoglobinopathy patients can be listed as follows: 1) State of high cardiac output due to chronic hemolytic anemia and hypoxia; 2) Cardiac toxicity of accumulated iron due to chronic transfusions and ineffective erythropoiesis; 3) Micro- or macrovascular pathologies seen as a result of endothelial dysfunction, increased arterial stiffness, decreased nitric oxide, and a hypercoagulable state; 4) Valvular disease due to high output and elastic tissue deficits [2,3,6,7].

The relevant cardiac manifestations, associated conditions, and pathophysiological mechanisms are presented in Table 1.

For hemoglobinopathy patients regularly followed by hematology units with no cardiological pathology, regular annual cardiological evaluations are necessary. Anamnesis and physical examination are the basis of these evaluations and electrocardiogram (ECG), echocardiography (ECHO), chest radiography, and, if necessary, biomarkers can be considered as basic clinical tests [8].

T2* sequence cardiovascular magnetic resonance imaging (MRI) is another method that has become increasingly used in recent years. It reveals myocardial iron load with high sensitivity. T2* times of over 20 ms as measured by 1.5-T MRI devices are considered normal with no cardiac iron overload, T_2_* times between 10 and 20 ms reflect mild to moderate iron load, and T_2_* times of <10 ms reflect severe iron overload [9]. In the evaluation of hemoglobinopathy patients, if no problems are observed in the basic cardiological evaluation and tests and there are no clinical findings, the patient should be evaluated annually by a cardiologist. If MRI is performed with the suspicion of iron overload and T2* is found to be <20 ms, the patient should undergo cardiological evaluation every 6–12 months. In the event of any newly developed symptoms, cardiological evaluations should be repeated as necessary [3,8,10]. In addition to annual cardiological evaluations in patients with transfusion-dependent hemoglobinopathy, baseline T2* MRI evaluations should be performed if possible. If the T2* MRI results are normal, these patients should be reevaluated every 2 years, while if there are signs of T2* of <20 ms or heart involvement, T2* MRI should be performed within 1 year at latest [3,8,10].

## 3. Immunoglobulin light chain-associated amyloidosis and the heart

Amyloidosis is a group of diseases that cause different organ pathologies as a result of the accumulation of insoluble pathological amyloid fibrils in many organs including the heart, kidney, liver, skin, spleen, and nerves. The most common form of amyloidosis is immunoglobulin light chain-associated (AL) amyloidosis. AL amyloidosis is an acquired condition that originates from a clonal plasma cell population producing misfolded amyloidogenic light chains. The mechanism of tissue damage in amyloidosis has been explained by oxidative stress, inflammation, induction of apoptosis, and direct toxic effects [11]. The median age at disease diagnosis is 76 years with a male predominance. Although the most commonly involved organ is the kidney, the most important organ involvement that determines prognosis is involvement of the heart [11–14]. AL amyloidosis, also known as primary amyloidosis, is a rare disease with an incidence of 3–5 per 1,000,000 [14].

It is important to note that the diagnosis process is often delayed in cases of this disease, which is known to entail life expectancy of less than 6 months in untreated cases. The most common symptoms of amyloidosis are fatigue and dyspnea. The diagnosis may be delayed due to these nonspecific symptoms and other findings that mimic those of many other diseases. Dyspnea is the most important of the main symptoms of cardiac involvement. Cardiac amyloidosis presents with limited cardiomyopathy or heart failure with preserved ejection fraction, characterized by right-sided heart failure. In addition, arrhythmias such as atrial fibrillation and conduction blocks may be observed in patients with cardiac amyloidosis, and hypotension, syncope, or autonomic neuropathic findings may also be present [12,14,15]. In patients with smoldering multiple myeloma or monoclonal gammopathy of undetermined significance, amyloid development may occur without typical end-organ damage or myeloma transformation. In this patient group, hematologists and, if referred, cardiologists and other relevant physicians (nephrology, gastroenterology, neurology, etc.) must be prepared to interpret the findings appropriately and prevent the delayed diagnosis of amyloidosis. Diagnosis can be performed for these patients with laboratory tests after anamnesis and physical examination. In patients with monoclonal gammopathy, the development of proteinuria, the presence of neuropathy, autonomic dysfunction, and heart failure with preserved ejection fraction are warning signs of amyloid development. The tests and diagnostic algorithm required to screen for AL amyloidosis are presented in Figure.

If the serum and urine immunofixation results are negative and the serum free kappa/lambda ratio remains within the range of 0.26–1.65, the probability of AL amyloidosis is low. The differential diagnosis of other types of amyloidosis should be considered. In such cases, a biopsy of appropriate tissues and/or cardiac imaging with technetium for transthyretin-associated amyloidosis (ATTR) should be performed to establish a clear differential diagnosis [11,13,14].

Four criteria have been defined for the diagnosis of AL amyloidosis and all four criteria must be met for a diagnosis [16,17]: 1) Evidence of organ damage associated with amyloid deposition, not due to any other conditions; 2) Detection of the presence of amyloid in tissues; 3) Confirmation that the amyloid is light chain-related; and 4) Evidence of monoclonal plasma cell proliferation.

For patients with a confirmed diagnosis of AL amyloidosis, ECG, ECHO, and B-type natriuretic peptide (BNP), N-terminal pro-brain natriuretic peptide (NT-pro-BNP), and cardiac troponin T/I (cTnT/I) measurements should be performed at baseline to evaluate cardiac involvement, disease staging, and prognosis.

ECG findings of cardiac amyloidosis are low voltage in the extremity leads, pseudoinfarction-pattern Q waves, weak R wave voltage increase in the V1–V3 leads, and AV conduction defects. These ECG findings may be present for all types of amyloidosis and none of them are uniquely specific to AL cardiac amyloidosis.

The ECHO findings of cardiac amyloidosis are not type-specific, similarly to ECG findings. The ECHO findings of amyloidosis are as follows: thickness of the interventricular septum and posterior wall of the left ventricle ≥12 mm; granular sparkling of the myocardium; increased valve thickness; reduced longitudinal strain with apical sparing pattern; size decreases of the ventricles and increase in size of the atria; and pericardial effusion.

Diffuse subendocardial late gadolinium enhancement is a typical pattern on MRI in amyloid patients with cardiac involvement. The characteristic pattern of AL cardiac amyloidosis is diffuse subendocardial late gadolinium enhancement. Since the gadolinium distribution in AL cardiac amyloidosis is not compatible with the blood area of the coronary arteries, the MRI finding is distinctive from that of ischemic cardiomyopathy. Although there are features that distinguish AL amyloidosis from ATTR with radiological mapping, the type of disease cannot be clearly differentiated based on MRI findings alone [12].

BNP, NT-pro-BNP, and cTnT/I levels are laboratory parameters used in staging, prognosis, and response evaluation in cases of AL cardiac amyloidosis. There are different scoring systems developed for staging. The Mayo 2012 system, shown in Table 2, is the most commonly used staging system in both daily practice and clinical research [18].

The presence of Congo red-positive deposits of amorphous material in biopsy findings is the gold standard for the diagnosis of amyloidosis. Once amyloid deposition is detected, amyloid subtype classification should be performed. Mass spectrometry, immunohistochemistry, or immunoelectron microscopy can be used for classification [15]. Since the biopsy area poses challenges in cardiac amyloidosis, noninvasive tests should be performed first, but in cases where a clear diagnosis cannot be made, endomyocardial biopsy should be considered. In AL cardiac amyloidosis, there is no need for cardiac biopsy because the amyloid will be detected in other tissues. Noninvasive tests will be sufficient with the features described above.

There have been many new treatment developments for AL amyloidosis in recent years. Despite this trend, however, there is still no clear curative treatment. With the introduction of CD38-targeted therapies, treatment modalities are now available for some patient groups without autologous stem cell transplantation [19–21]. Clinical research has been conducted recently for adding anti-amyloid fibril antibody treatment to standard AL amyloidosis treatments [20,22]. Although deep responses are seen with these treatments, relapse is common. Cardiac involvement plays the main role in treatment modality and treatment response evaluation. Considering other organ involvement, AL amyloidosis treatment should be continued with a multidisciplinary approach. In patients with cardiac involvement, during the initial hematological treatment, a monthly clinical evaluation, complete blood count, basic biochemistry analysis, NT-pro-BNP and cTnT/I measurements, serum free light chain quantification, and, if necessary, cardiological evaluation are required. Hematological clinical evaluation, complete blood count, basic biochemistry analysis, NT-pro-BNP and cTnT/I measurements, serum free light chain quantification, and, if necessary, cardiological evaluation are recommended every 3 months for patients who are followed after maintenance treatment or autologous transplantation. Even if there is no clinical progression, for every AL amyloidosis patient with cardiac involvement a cardiologist’s evaluation with ECG, ECHO, or MRI every 6 months and follow-up with Holter ECG every 12 months would be appropriate [14,23].

## 4. Myeloproliferative diseases and the heart

Myeloproliferative diseases are clonal hematopoietic conditions including three main entities: polycythemia vera, essential thrombocytosis, and primary myelofibrosis. The Janus kinase 2 (*JAK2*), calreticulin (*CARL*), and myeloproliferative leukemia protein (*MPL*) genes shape the basic pathological mechanism. Driver mutations and other mutations such as those of the tet methylcytosine dioxygenase 2 (*TET2*), DNA methyltransferase 3 alpha (*DNMT3A*), and additional sex Combs-like 1 (*ASXL1*) genes have been associated with systemic inflammatory status [24]. Clonal hematopoiesis of indeterminate potential (CHIP), defined as the precursor status of myelodysplastic syndrome, entails many clonal mutations including *TET2*, *DNMT3A*, and *ASXL1*. Although *JAK2*V617F is the mutation most strongly associated with cardiovascular disease risk and thrombosis, most of the mutations seen in cases of myeloproliferative neoplasm (MPN) and CHIP were found to affect the development of cardiovascular disease in many studies [24,25]. The rate of cardiovascular involvement has been shown to vary between 4% and 28% in MPN patients [24,26]. Cardiovascular diseases are the main cause of mortality and morbidity in these patients. Cardiovascular system involvement in MPN patients may present with clinical findings such as arterial or venous thrombosis, accelerated atherosclerosis, heart failure, and pulmonary hypertension. It has been shown that cardiovascular involvement in MPN and CHIP patients is associated with an increase in inflammatory cytokines; an increase in lysyl oxidase, an enzyme that plays a role in collagen and elastin fibril maturation; leukocytosis; thrombocytosis; erythrocytosis; erythrocyte anisocytosis; and renin–angiotensin system changes associated with JAK–STAT pathway activation [24]. After the diagnosis of MPN, the patient’s risk class should be clarified before beginning treatment. Age over 60 years and/or presence of thrombosis is an indicator of high risk in MPN patients and cytoreductive treatment is required for this patient group. MPN patients should be evaluated in terms of thrombotic processes and cardiovascular risk classes, as those will affect the choice of treatment.

It was shown in a prospective study that nonfatal myocardial infarction, nonfatal stroke, pulmonary embolism, major venous thrombosis, and death from cardiovascular causes are reduced by 60% with acetylsalicylic acid (ASA) in polycythemia vera patients [27]. Therefore, ASA should be given to polycythemia vera patients at 81–100 mg per day regardless of risk classification unless there is a contraindication [28].

There are no randomized controlled studies showing that the risk of thrombosis decreases with the use of low-dose ASA in patients with essential thrombocytosis. Since it has been concluded from retrospective data that *JAK*-negative essential thrombocytosis patients under the age of 60 who are classified as very low risk and who have no history of thrombosis or cardiovascular disease can be monitored without ASA, these patients may not be given ASA [28–30]. However, all MPN patients should be offered lifestyle modifications for cardiovascular risk reduction. These suggestions may include healthy nutrition, weight control, quitting smoking or alcohol, adequate and appropriate physical activity, avoiding stress, adequate fluid intake, blood pressure control, and the control of other comorbid conditions. For patients who develop thrombosis during follow-up, low-molecular-weight heparin, direct oral anticoagulants, and warfarin can be used depending on the location of the thrombosis and the characteristics of the patient. ASA may be appropriate for patients with cardiovascular risk factors. There are no clear recommendations regarding the duration of treatment and the coadministration of ASA or antiplatelet therapy with anticoagulant therapy. Thus, the risks and benefits of aspirin plus anticoagulation and the duration of the therapy need to be considered on an individualized basis.

## 5. Other hematological conditions and the heart

Hypereosinophilic syndrome (HES) is a rare spectrum of disease diagnosed based on an eosinophil count above 1500/μL in peripheral blood and the exclusion of secondary and clonal eosinophilia. It is characterized by findings of organ damage and dysfunction mediated by eosinophilia. Cardiovascular complications are responsible for the significant morbidity and mortality rates associated with HES. In addition to direct eosinophil-mediated damage to the heart, coronary vasospasm, arterial and/or venous thrombus, and microvascular damage may also occur in HES. Cardiac involvement due to eosinophilia has been reported in 20% of HES patients [31].

Progressive heart failure is the most typical clinical outcome of eosinophilic organ damage. The direct eosinophilic infiltration of tissue and the release of toxic substances are responsible for this situation. Endocardial damage leading to the formation of platelet aggregates can cause mural thrombus formation and embolism. In the advanced stage, endocardial restrictive cardiomyopathy may develop as a result of fibrosis. Mural endocardial thrombosis and fibrosis affect the mitral and tricuspid valves, leading to valvular failure.

Cardiovascular follow-up is also important in the evaluation of cardiac involvement and treatment response, which is very important in the prognosis of HES. The initial approach to the cardiac evaluation of a patient with HES should be thorough anamnesis and a comprehensive physical examination for cardiovascular involvement. Baseline ECG, ECHO, and chest X-ray should be obtained. ECG findings are usually nonspecific, such as nonspecific S-T and T wave abnormalities, bundle branch blocks, left atrial enlargement, left ventricular hypertrophy, ventricular premature complexes, and left axis deviation [32,33]. ECHO findings in HES include endomyocardial thickening, left and right ventricular apical thrombus, posterior mitral leaflet involvement, regurgitation of atrioventricular valves, and pericardial effusion [32,33]. Cardiac MRI is more sensitive and specific than ECHO in detecting ventricular thrombosis. Contrast-enhanced imaging can detect inflammation and myocardial fibrosis. Despite the existence of many noninvasive methods for evaluating cardiac involvement in HES, endomyocardial biopsy is the diagnostic gold standard to demonstrate pathological involvement. It should be performed carefully and only when necessary [32,33].

In hematological malignancies, both disease progression and treatment-related cardiovascular events can occur. As a result of a very large metaanalysis that included 1,960,144 cases published very recently, patients with hematological malignancies were shown to be at higher risk for the development of acute myocardial infarction, heart failure, and stroke than those without malignancies [34].

Extramedullary cardiac involvement in cases of malignant hematological diseases such as lymphomas, leukemias, and multiple myeloma is extremely rare. Primary cardiac lymphoma is also extremely rare, accounting for 1.3% of all primary cardiac tumors [35] and 0.5% of all lymphomas [35].

Compared to primary cardiac lymphoma, secondary cardiac lymphoma involvement is more common among lymphomas. ECHO is frequently used to visualize cardiac hematological tumors. In cases where there is difficulty in obtaining results by ECHO, computed tomography and/or MRI may be preferred.

The most important factor that causes cardiac issues in hematological diseases is drug administration. Drug-related cardiomyopathies, arrhythmias, and vascular problems can adversely affect the treatment course and therefore the prognosis of the patients.

Drug-induced cardiotoxicity is a side effect seen with many drugs used in the treatment of hematological diseases, especially anthracyclines, tyrosine kinase inhibitors, and immune checkpoint inhibitors. They cause clinical findings due to the functional and structural changes that occur in the cardiovascular system due to direct cardiac toxicity as well as possible overdose or cumulative drug toxicity. These clinical findings include heart failure, myocardial ischemia, valve disease, thrombosis, myocarditis, pericarditis, arrhythmias, and conduction abnormalities [36,37]. The disruption of ionic processes, cellular damage due to mitochondrial dysfunction, hypercoagulability, and immunological effects have been defined as mechanisms of drug-related cardiotoxicity [36,38,39]. It should be kept in mind that, theoretically, every drug has the ability to affect the functioning of every organ system. Since many postmarketing side effects of drugs developed as a result of clinical research have been identified, even if they have not been previously identified in studies of the drug, effects that cannot be attributed to another cause that resolve after discontinuation of the drug and are triggered upon restarting the drug should be suspected as side effects and the necessary pharmacovigilance notifications should be made. Baseline evaluations of the most well-known side effects are important before starting drug therapy for a patient. Drugs frequently used in hematology with known cardiotoxicity, their toxicity, and the evaluations that should be conducted before beginning therapy are presented in Table 3.

## 6. Conclusion

The present review was conducted based on widely accepted theoretical knowledge and the latest published literature. Many hematological diseases and treatments are closely associated with the cardiovascular system. Similarly, some hematological diseases may present with cardiac manifestations. Such involvement should be reviewed at each follow-up appointment and closely monitored. Cardiac involvement or presentations are an important issue for hematological patients. Multidisciplinary evaluation of patients should be performed in hematology practice and in cases of cardiac involvement.

## Figures and Tables

**Figure:**
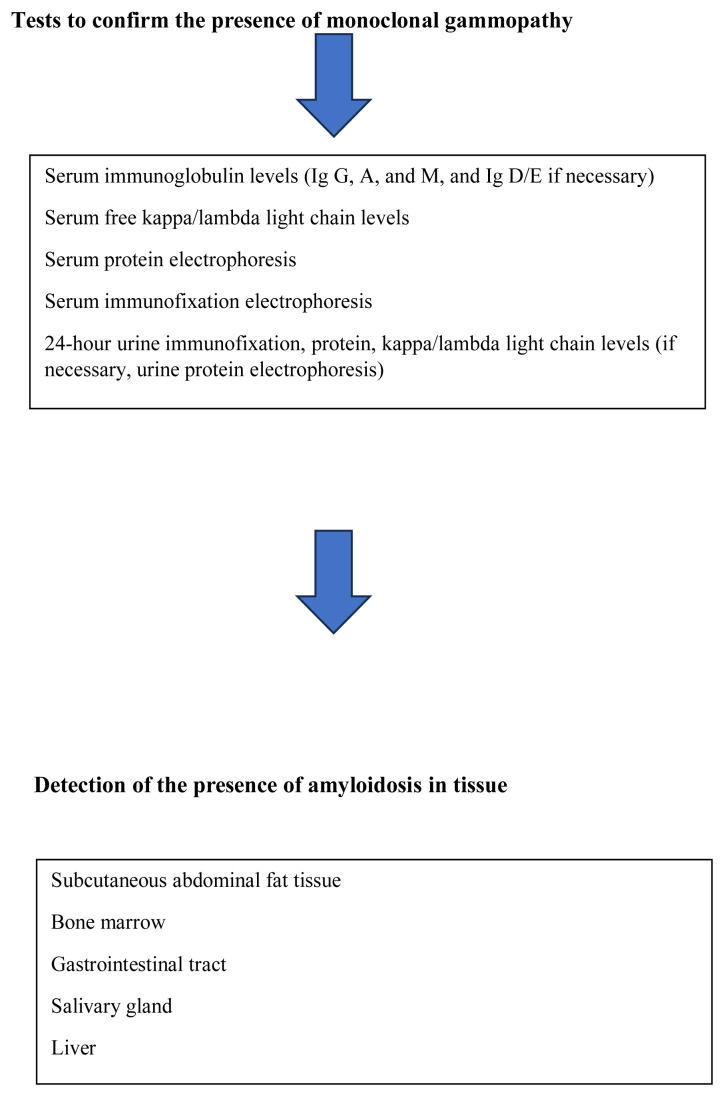
Diagnostic algorithm for AL amyloidosis.

**Table 1:** Cardiac manifestations, associated conditions, and pathophysiological mechanisms of hemoglobinopathies.

Clinical manifestations	Conditions	Causes
Arrhythmias	AF, supraventricular tachycardia, ventricular tachycardia, ventricular fibrillation	Iron overload
Left ventricular dysfunction		High output state, iron overload, vascular disease, myocardial ischemia, myocarditis, valvular disease
Myocarditis		Iron overload, infections, immune dysregulation
Myocardial infarction Angina		Vasculopathy (endothelial dysfunction, occlusion, inflammation), myocardial fibrosis
Pericarditis	Acute pericarditis, chronic constrictive pericarditis	
Pulmonary hypertension		High output state, left ventricular dysfunction, vasculopathy, lung disease
Right ventricular dysfunction		High output state, iron overload, left ventricular dysfunction, pulmonary hypertension, vasculopathy, ischemia, valvular disease
Valvular pathologies	Valvular calcifications, mitral valve prolapse, mitral regurgitation, aortic stenosis	High output state, elastic tissue defects
Vascular disease	Macrovasculopathy, microvasculopathy	Endothelial dysfunction or defect, increased arterial stiffness, hypercoagulable state, decreased NO

AF: Atrial fibrillation; NO: nitric oxide.

**Table 2:** Mayo 2012 AL amyloidosis staging system and prognosis.

Stage	Risk factors	Number of risk factors	Median OS (months)
I	cTnT of ≥0.025 μg/L or high-sensitivity cTnT of ≥40 ng/L NT-pro-BNP of ≥1800 ng/L or BNP of ≥400 ng/L dFLC of ≥18 mg/dL	None	94
II		1	40
III		2	14
IV		3 (all)	6

AL: Immunoglobulin light chain-associated; OS: overall survival; cTnT: cardiac troponin T; NT-pro-BNP: N-terminal pro-brain natriuretic peptide; BNP: brain natriuretic peptide; dFLC: difference between involved and uninvolved serum free light chains.

**Table 3:** Cardiotoxic drugs using in hematology practice.

Drug type	Mechanism	Dosage relation or association	Clinical presentation

Alkylating agents (cyclophosphamide, cisplatin, ifosfamide)	Myocardial ischemia, endothelial cell injury, cisplatin-related toxicity due to the volume of administration, arterial hypertension	Concentration-dependent*	Acute ischemic events, myocardial infarction, ischemic cardiomyopathy, arrhythmias, heart failure, pericardial effusion

Anthracyclines (adriamycin-doxorubicin, idarubicin, daunorubicin, epirubicin, mitoxantrone)	Myocardial injury due to oxidative stress and development of fibrosis	Cumulative dosage-related**	LV systolic dysfunction, arrhythmias

Arsenic	Not clear		QTc interval prolongation, torsade de pointes, heart failure

Fluoropyrimidines ( fluorouracil, capecitabine )	Arterial vasospasm, endothelial cell injury, thrombosis, myocarditis due to direct myocardial toxic effect of drug’s metabolites	Dosage-independent but administration-dependent***	Angina, myocardial infarction, arrhythmias, pulmonary edema, cardiac arrest, pericarditis

Histone deacetylase inhibitors (romidepsin, vorinostat)	Not clear		ECG changes, arrhythmias (especially QTc prolongation)

Immune checkpoint inhibitors for PD1/PDL1 and CTLA4 (ipilimumab, nivolumab, pembrolizumab, etc.)	Tissue inflammation and damage		Myocarditis, myositis, heart failure, pericarditis, heart block, arrhythmias, myocardial fibrosis, cardiomyopathy

Proteasome inhibitors (bortezomib, carfilzomib)	Not clear		Acute pulmonary edema, cardiac failure, cardiogenic shock, hypertension

Tyrosine kinase inhibitors	Inhibition of K^+^ channel and prolonged cardiac ventricular repolarization		Cardiac dysfunction, pericardial effusion, arrhythmias, QT prolongation, hypertension, ischemic heart disease Atrial flutter and fibrillation, QTc shortening, hypertension, heart failure, myocardial infarction, ventricular arrhythmias, sudden cardiac death QTc prolongation, heart failure Hypertension, ischemic heart disease, arrhythmias, QTc prolongation Vascular occlusion, hypertension, left ventricular dysfunction, QTc prolongation, arrhythmias ECG changes, QTc prolongation, arrhythmias, hypertension
Dasatinib			
Ibrutinib, acalabrutinib	Not clear		
Imatinib	Impaired cell signal transduction		
Nilotinib	Prolonged cardiac ventricular repolarization		
Ponatinib	Not clear		
Sorafenib	Inhibition of RAF1, PDGFRs, and VEGF receptors		

Vinca alkaloids (vincristine, vinblastine)	Vascular toxicity, hypoxia		Myocardial ischemia, hypertension

* High-dosage protocols (>50 mg/kg) have more toxicity;

** Cumulative dosage threshold of >90 mg/m^2^ for idarubicin, 900 mg/m^2^ for epirubicin, 120 mg/m^2^ for mitoxantrone, 400–550 mg/m^2^ for doxorubicin, 250 mg/m^2^ for daunorubicin;

*** Higher risk with intravenous infusion compared to bolus administration.
